# Generation and Implementation of a Patient-Centered and Patient-Facing Genomic Test Report in the EHR

**DOI:** 10.5334/egems.256

**Published:** 2018-06-26

**Authors:** Jessica M. Goehringer, Michele A. Bonhag, Laney K. Jones, Tara Schmidlen, Marci Schwartz, Alanna Kulchak Rahm, Janet L. Williams, Marc S. Williams

**Affiliations:** 1 Genomic Medicine Institute, Geisinger, Danville, PA, US; 2 Patient Co-investigator, Chase Group, Ltd, Traverse, Michigan, US; 3 Center for Pharmacy Innovation and Outcomes, Geisinger, Danville, PA, US

**Keywords:** genomics, electronic health records, patient-centered care, patient access to records, communication

## Abstract

**Context:**

Communication of genetic laboratory results to patients and providers is impeded by the complexity of results and reports. This can lead to misinterpretation of results, causing inappropriate care. Patients often do not receive a copy of the report leading to possible miscommunication. To address these problems, we conducted patient-centered research to inform design of interpretive reports. Here we describe the development and deployment of a specific patient-centered clinical decision support (CDS) tool, a multi-use patient-centered genomic test report (PGR) that interfaces with an electronic health record (EHR).

**Implementation Process:**

A PGR with a companion provider report was configured for implementation within the EHR using locally developed software (COMPASS™) to manage secure data exchange and access.

**Findings:**

We conducted semi-structured interviews with patients, family members, and clinicians that showed they sought clear information addressing findings, family implications, resources, prognosis and next steps relative to the genomic result. Providers requested access to applicable, available clinical guidelines. Initial results indicated patients and providers found the PGR contained helpful, valuable information and would provide a basis for result-related conversation between patients, providers and family.

**Major Themes:**

Direct patient involvement in the design and development of a PGR identified format and presentation preferences, and delivery of relevant information to patients and providers, prompting the creation of a CDS tool.

**Conclusions:**

Research and development of patient-centered CDS tools designed to support improved patient outcomes, are enhanced by early and substantial engagement of patients in contributing to all phases of tool design and development.

## Context

Genetic disorders, while individually rare, are collectively common. It is estimated that there are over 6,800 rare and ultra-rare disorders, many of which are genetic, affecting approximately 30 million Americans [1]. The challenge for patients and their providers is having ready access to necessary information for appropriate management and coordination of care. When faced with a patient with a genetic condition they have not previously encountered, lack of such access may preclude the best care practices. This often puts patients and families in the position of attempting to become the ‘expert’ in the specific disease via an unguided internet search process. McMullan [2] indicates that health care professionals respond to patients with such internet-acquired expertise in one or more ways, including feeling threatened by the information and responding defensively, by collaborating with the patient and forming a patient-centered relationship, or by helping to guide the patient to the most reliable health information. One possible solution to foster the second and third scenarios above in the context of genomic medicine is to utilize the power of a fully functional electronic health record system (EHR) [3,4,5]. The capabilities provided by such EHRs, particularly through clinical decision support (CDS) systems, have been demonstrated to significantly improve certain care processes, although the evidence of impact on health outcomes such as morbidity or mortality is less robust [6].

While these CDS efforts have been primarily provider-centered, similar approaches could be used to provide information directly to patients and families. A recent study conducted at three health care organizations, including Geisinger, studied the impact of opening patient access to their provider notes through a secure patient portal (OpenNotes) [7]. Using a quasi-experimental trial design, several significant differences were noted when comparing the pre- and post-intervention surveys including patients reporting that OpenNotes, “…helped them feel more in control of their care” and “…increased medication adherence.” A recent report noted that patient-facing applications could enable meaningful use objectives promoted by the Office of the National Coordinator of Health Information Technology (ONCHIT) [8]. This approach is also endorsed by the recent report from the National Academy of Medicine (formerly the Institute of Medicine), *Improving Diagnosis in Health Care* [9], which highlights the importance of patient empowerment.

An important implication of open access includes the patient’s ability to view laboratory reports. More recently, the Department of Health and Human Services determined that laboratories must make reports accessible for patients who request them. The genomic laboratory report could represent a possible conduit for the transmission of important clinical information to patients. Traditionally, genetic laboratory reports are intended for clinicians with the assumption that these clinicians have their patient’s medical history and the required knowledge to interpret the results [10]. As such, current genetics laboratory reports are not patient-friendly. Haga [11] proposes four revisions to current genomic test report formats that could improve accessibility for patients. These include, “…1) inclusion of an interpretive summary section, 2) a summary letter to accompany the laboratory report, 3) development of a patient user guide to be provided with the report, and 4) a completely revised patient-friendly report.” A commercial example of genomic result reports following these recommendations are now available in the direct-to-consumer genetic testing market. These reports, designed for consumers without the involvement of providers, convey predictive information based on test findings. While these reports do not use EHR-based clinical decision support, they are early examples of communication vehicles for deliver complex genomic information to consumers. This approach to communication contributed to the recognition of the potential for a laboratory genomic results report to directly inform the lay public.

Genome and exome sequencing have prompted new gene/disease recognition, particularly in the realm of intellectual disability (ID) and autism spectrum disorder (ASD). Etiologies for ID and ASD are increasingly identified to be caused by specific genetic findings. The challenge for patients and their providers is having ready access to the information that is necessary for appropriate management and coordination of care. These new diagnostic results often have no existing treatment protocols leaving providers, patients and their families to try to create relevant management and treatment plans. Another challenge patients and providers may face is the burden of secondary genomic results, based on the recommendation for laboratories to report medically actionable variants found within 59 genes, regardless of the reason for testing [12]. With common disease, such as *BRCA1/2*-related cancer, evidence-based professional practice guidelines allow providers access to best practices that can inform shared decision making with their patients. For many genetic conditions, including ID and ASD, there are few practice guidelines. This impacts non-genetic providers who may not recognize the condition or the gene reported and may not have resources readily available to inform appropriate care decisions.

We hypothesized that a new form of functional genetic test report, presented electronically through an EHR with a patient view, could dramatically improve the shared decision-making and condition-specific management and improve outcomes from both the patient/family and provider perspectives. Therefore, in order to develop a complex genomic lab report accessible, understandable, and usable by the patient, we recognized the need to secure active involvement by patients. From the outset, a patient investigator joined the team of other investigators and contributed to the development of the genomic results proposal for submission to the Patient Centered Outcomes Research Institute (PCORI). She has since participated in all discussions, meetings, evaluations, analysis, writing of publications, and has spoken in multiple venues about being a member of the research team.

Here we describe patient partnership in the research that developed and tested a patient-centered CDS genomic reporting tool designed to improve communication and understanding of genomic sequencing results for patients and providers.

## Case Description/Implementation Process or Intervention

### Setting

Geisinger is a rural, integrated health care delivery system in central Pennsylvania and southern New Jersey, serving approximately 4.2 million residents with about 1.5 million unique patient visits annually. Geisinger is partnering with the Regeneron Genetics Center to perform exome sequencing on as many as 250,000 Geisinger patients over the next 5 years [13]. Geisinger is recognized for informatics support for clinical care and represented an ideal place to research and build a patient centered genomic results report (PGR).

### Methodology: Initial Use Case-Whole Genome Sequencing for children with undiagnosed ID/ASD

**Figure 1:**
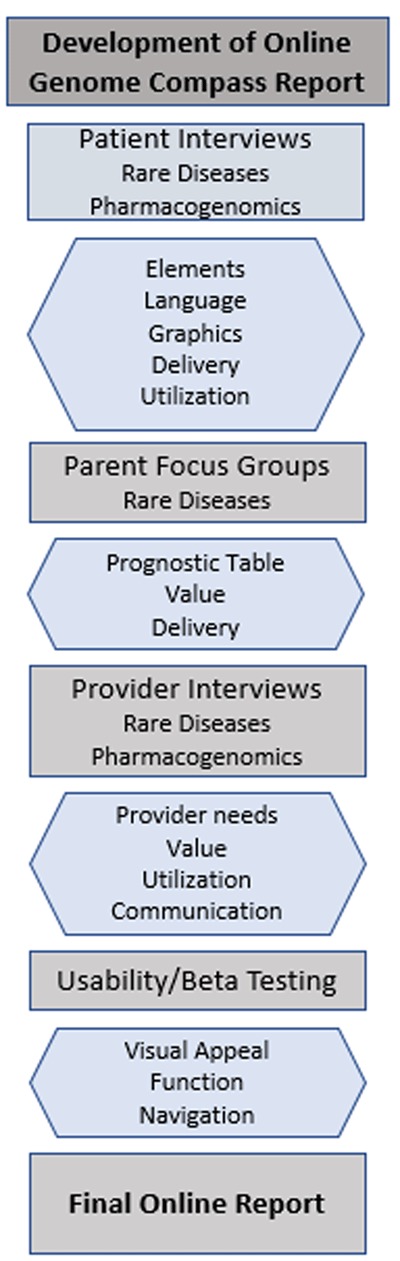
Flow diagram of each phase involved in the development and deployment of the PGR. Phase 1 consisted of qualitative interviews and focus groups with parents of children in WGS clinical research study. This lead to physician interviews to elicit their thoughts on the PGR. Phase 2 focused on the development and testing of the online version of the PGR. The third research phase, outlined in more detail in Figure 2, utilized a prospective mixed-methods study of the online PGR to evaluate access, use and satisfaction.

At the initiation of a clinical research study to offer whole genome sequencing to children with undiagnosed ID and ASD, researchers realized that communication of the results, which could include both primary and secondary genomic sequence findings, represented a research opportunity to study an innovative type of results communication (Figure 1). A protocol was written and submitted to study the effectiveness of a PGR on patient-centered outcomes focused on communication, engagement and satisfaction, using a prospective randomized pre-post asymmetric crossover study paired with post-intervention qualitative evaluation [14,15]. The study was approved by the Geisinger Institutional Review Board, registered at clinicaltrials.gov (Record 2013-0594), and funded by PCORI (CD-1304-6987).

An investigator team was formed and included experts in genomics, qualitative research, informatics, health literacy, consumer advocacy and importantly, a patient investigator. A mixed-methods study design utilized three phases for research. The initial phase consisted of qualitative interviews and focus groups with parents of the children enrolled in the whole genome sequencing (WGS) clinical research study.

The parents were asked to review and comment on a paper copy of a sample genomics results report based on the report previously designed by members of the team in response to the CLARITY Challenge [16]. Participants were asked to review the elements, language and format as well as to comment on what was missing or what constituted too much information. There was a section of the report that parents nearly unanimously requested be further developed. This led to focus groups with parents (some of whom had provided opinions in the first round) who were asked to help develop the section on “Next Steps” and “Prognosis” with information related to the genomic finding on the report. Analysis of the qualitative data of both the interviews and the focus groups led to a formalized patient genome results report [10].

Subsequent to the creation of the PGR, a small convenience sample of physicians from Geisinger, without any formal genetics training, were asked to participate in interviews to elicit their perspectives on the genome results report [17]. A single interviewer conducted semi-structured interviews with six providers using an interview guide to obtain feedback on each section of the PGR in a stepwise manner.

Utilizing the perspectives provided by patients and providers regarding key elements in a results report, the next phase focused on the development and testing of an online functional version of the PGR. Researchers adapted a Geisinger-developed software platform, COMPASS™, previously created to manage secure data exchange with access to patient reported data as well as capture EHR data to run CDS and display the results to clinicians. COMPASS™ was widely integrated with Geisinger’s primary EHR (Epic) in several clinical settings and the patient portal, which made it a good vehicle for the functional PGR version.

The COMPASS™ tool includes an authoring application where providers, geneticists and genetic counselors in this developmental study, build coordinated and complementary patient-specific genomics results reports. The authoring application pulls relevant patient-specific information from the EHR. The patient’s genomic sequencing result (i.e., the gene and genomic variant) must then be manually entered; however, the genomic sequencing original laboratory report is linked to the provider version of the PGR in the EHR. The application was created to include a data bank of reusable content fields including the gene specific template, glossary terms, inheritance patterns, patient resources and provider resources. The environment can track PGR versioning, an essential element given the rapidly changing nature of knowledge in genomics. For this study, an interface involving an additional CDS diagnostic tool, SimulConsult Genome-Phenome Analyzer (SGPA) [18] provided additional functionality that our formative research identified as highly desirable by patients and providers. Upon selection and verification of the content, the report is then published from the authoring application and the final patient-specific PGR becomes available through COMPASS™ in the MyGeisinger patient portal. (This report is also accessible to clinicians in the patient’s EHR through a local Web Apps URL.) The final step in this phase involved usability testing for feedback on clarity and ease of navigation.

**Figure 2:**
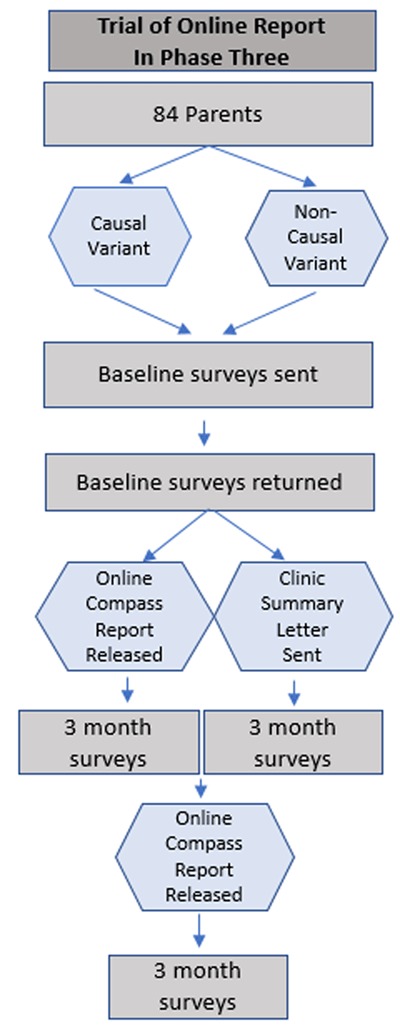
Flow diagram of Phase 3 (randomized mixed-methods descriptive study on online PGR).

The third research phase (Figure 2) consisted of a prospective randomized mixed-methods descriptive study of the online PGR to evaluate access, use, and satisfaction with the report. The study population included 42 parent-couples (N = 84) of children enrolled in the WGS study noted above. Parents were the appropriate participants for this study as they have the primary responsibility for the care and management of their child(ren) with these chronic, rare conditions and are the recipients of communication of sequencing test results. As part of the WGS study, all parents participated in a traditional genetics clinic visit to learn of the results of the WGS study for their child. After the clinic visit, parents were stratified by their child’s result (diagnostic vs. uninformative) and then randomized into an intervention arm in which they received notice via MyGeisinger message of access to the COMPASS™ PGR or to the usual care arm in which a standard genetics visit summary letter was sent by mail. Both parents and investigators were blinded to the randomization. All parents were individually sent an invitation letter that included a baseline survey designed to collect demographic information, measure comfort with health information [19], health literacy [20], decision regret [21] (relative to participation in the WGS study) and satisfaction. Enrollment in this phase was determined by return of the baseline survey. Once the baseline survey was returned, those parents assigned to the intervention arm received access to the PGR and those assigned to the usual care arm received their summary letter in the mail. At three months post release, surveys were sent to measure change in the participant’s responses from baseline. For those with a diagnostic result, the PAGIS [22] scale was added to measure understanding related to the new genetic information. An asymmetric crossover arm released the PGR to all enrolled participants who were in the usual care arm. At 3 months post release, those participants also received the post-PGR survey. Interviews were completed with participants who failed to follow through with survey completion as well as with those who had diagnostic results vs those who received noninformative results to learn more about their experience with the online PGR [15].

The data collected from the surveys included demographics, use of the EHR, use of resources, and a set of validated instruments including satisfaction, decision regret and other outcomes. One genetic counselor highly skilled in qualitative research conducted the semi-structured interviews, using an interview guide, at three months post-intervention to further understand outcomes including utilization, impact, and unintended consequences of the enhanced genomic report [15]. Parent participants were asked to provide feedback on areas of focus including potential improvements to the Family Report, success in communication, and unmet needs related to the communication of genetic information [10]. Thematic saturation was reached after the eighth individual participant.

### Methodology of Additional Use Cases: Pharmacogenomics and population exome sequencing

The lessons learned through patient involvement in the development of the COMPASS™ genomic results report were next applied to design two new types of genomic reports, one to communicate pharmacogenomic (PG×) test results to patients, providers, and pharmacists [23], a second for genomic results reported to patients enrolled in the Geisinger MyCode® Community Health Initiative (MyCode) [13] who receive genomic results from whole exome sequencing of the patient-participants [24].

Given the distinct difference between PG× genetic results versus those typically returned for indicated clinical testing for a condition, it was determined that a formal approach using the same engagement methodology outlined above was needed to develop the COMPASS™ PG× report. Adult MyCode patients and Geisinger clinicians were invited to participate in the study to develop the PG× report. Patients were identified using a random convenience sample and had to agree to drive to our research location in Danville, PA. Clinicians consisted of primary care providers, specialists and pharmacists who had participated in MyCode in different capacities. A total of ten patients, three PCPs, three specialists, and four pharmacists participated in in-person semi-structured interviews to review and comment on several versions of a mock PG× test report involving the gene *SLCO1B1*. Additional interviews were conducted with a fictional result involving the gene *TPMT* [23]. Interviews focused on the best vehicle, utility, timing, and EHR issues for reporting PG× results. This information was used to develop the COMPASS™ PG× report.

Results from the MyCode® whole exome sequencing were more similar to the results from the WGS project described above. As such, the existing report was modified by the development team (including the patient investigator) to reflect the information to be conveyed. Templates were created for each of the actionable genes for which pathogenic variants would be returned. Each of these templates was reviewed by patients to assess readability and content, as well as primary care providers and relevant specialists to ensure that the information included was clear, accurate and actionable.

## Findings

### Initial Use Case

Results of the analysis of the initial qualitative data obtained from the semi-structured parent interviews and focus groups support that participants desire clear and simple language about genetic testing results in a PGR (See Table 1). The key elements identified by parents included information about the gene(s), variant(s) and associated diagnoses, prognosis, information for other family members, a glossary of genetic terminology, and resources to disorder-specific support groups and sites designed for lay persons like Genetics Home Reference. To promote engagement and empowerment, and consistent with patient-centered outcomes research, our team opted to provide information about possible clinical trials relevant to the disorder (clinicaltrials.gov) and services such as GenomeConnect that support patient/family entered data to facilitate networking and research; parents approved this addition. Full details about the PGR are published elsewhere [10].

**Table 1:** Data collection methods and survey measures, utilized by outcome, to test impact of enhanced PGR and associated results.

Outcome	Collection Method	Measure	Timing of collection	Results

**Primary Outcomes**				

Utilization	COMPASS online report	Report accessed and by whom	3 month survey	33% of online reports accessed by parents or providers. Report accessed by those whose child had a diagnostic result found by genomic sequencing.
Satisfaction	Parent Survey	3 survey questions	3 month survey	Parents who received a diagnosis for their child reported satisfaction with the result. However those who did not receive a diagnosis for their child did not find a negative report helpful.
Impact	Parent Interview	Structured interview	3 months post-online report	Parents with a diagnostic result reported high understanding of the genetic information and ability to explain its implications to others.
**Demographics**				

Literacy	Parent Survey	Scale – HINTS	Baseline survey	High health literacy and Numeracy High overall information engagement subscale scores. Low information apprehension subscales scores.
**Numeracy**				

Race, marital status, education, employment	Parent Survey	Scale	Baseline survey	96% White; 88% Married; 63% Some college; 75% Employed.
**Secondary Outcomes**				

Decision Regret	Parent Survey	Scale – Decision Regret	Baseline, 3 months post- online report	90% of parents agree/strongly agree to no regret regarding their child’s participation in the whole genome sequencing research study.
Communication	Parent Interview	Online	Post-online report	Parents with a diagnostic result for their child saw value in sharing the report with other providers and care givers for their child.
Unintended Consequences	Parent Interview	Structured interview	3 months post-online report	Parents without a diagnosis for their child related that the negative result did not offer sufficient reason to access the online report.

Researchers were curious about which terminology to use in the report. Given the use of both incidental findings and secondary findings to describe results which may be found but are unrelated to the indication for the sequencing, we asked participants what they preferred. Parents suggested and preferred the use of “additional findings” for this type of result. Similarly, we asked participants about the use of pathogenic and likely pathogenic to describe positive findings and other medical terminology associated with symptoms. In this instance using “positive result” or “cause for your child’s symptoms” communicated more clearly the result of the test. Regarding terminology, they responded that we should include the medical terminology, include a glossary, and provide other simpler explanations as well. Parents told us this is because they need to know and recognize the medical language to understand what providers are saying and to be informed about their child’s condition.

Parents uniformly reported that they expected more detail and specific recommendations in the section labelled “Next Steps.” Initially this section listed recommended discussion topics to review with their health care provider. Parents indicated that since this is a report specific to their child’s result, there should be information available to provide prognostic information about what to expect in the future and specific actions to take as next steps; they wanted to have access to the same information that the “experts” know. Patients desired improved understanding of their child’s genomic sequencing result and assurance that health care providers would have access to accurate and current information about the result. In designing how to present this information, the patient investigator helped to shape the focus group process and was instrumental in determining the design options presented to the parents. The clinical team members considered one of the options to contain excessive information for patient use, but included it at the suggestion of the non-medical investigators and patient investigator. The focus group results, provider interviews, and post-randomized trial parent interviews supported inclusion of the detailed table as the most vital and valuable part of the PGR.

Our study also found that parents of children who received diagnostic results were more likely to access the PGR [15]. Parents and providers indicated that the enhanced PGR was a valuable tool and even demonstrated utility outside of the predicted realm of users; schools and therapists appreciated the clear, detailed content.

### Additional Use Cases

Patients who participated in the interviews to develop the PGR for PG× indicated they also desired a report that was simple, easy-to-understand, individualized, and provided information on how the PG× result influenced their current treatment plan. Patients reported after reading the report they felt better prepared to discuss with their health care provider how the PG× result could affect their current and future treatment plans [23]. Interestingly, these participants indicated that they expected that the report would be in their EHR and reviewed by their health care provider.

Providers indicated that they expect standard information such as demographics, clinical data and testing information. They also desired information such as indication for genetic testing (including key clinical findings of their patient), gene/variant findings, resulting primary and/or secondary diagnoses, a glossary of genetic terminology, and provider resources such as GeneReviews and Online Mendelian Inheritance in Man (OMIM). Providers requested access to the PGR so that they are aware of information provided to the patients and their families. Other groups have investigated the use of altered report formats to support clinical interpretation. As described by Gray [25], complex genomic data can confuse oncology providers and hinder the implementation of precision oncology. Gray’s team developed a provider-facing web-based interactive genomic report for somatic cancer sequencing with embedded clinician education and an optimized data layout. They utilized a randomized, vignette-based survey to determine whether their enhanced report improved clinician comprehension and satisfaction when viewing results of a somatic gene panel. They concluded that interactive genomic reports may support clinician-understanding of genomic data and increase report-related satisfaction [25]. Similarly in this study, providers reported they considered COMPASS™ genomic result report a CDS-tool that will assist patients and providers in understanding of and communication about rare disease information and management guidelines.

The deployment of the PGR encountered technical issues that were identified by participants as a barrier to using the report. When the PGR was launched for a patient, the message generated by the MyGeisinger portal was a default generic message that notified the recipient that a “survey” was available. This was confusing and led many patients to delete the message without accessing the report. A customized message was then developed to solve this issue. Another problem encountered involved access when the intended recipient was a caregiver of the patient. A caregiver requires proxy access to the MyGeisinger portal to receive the message and launch the PGR. Proxy access is now addressed during the clinical encounter when results are reported and caregivers are given instructions to create proxy access.

The most recent Geisinger application of the COMPASS™ genomic results report involves adapting the gene templates to enable reporting of genomic sequencing results that are part of the MyCode Community Health Initiative. At the time of this report, COMPASS™ genome reports have been released for 61 patient-participants and their providers. 20 patients have accessed the report. PG× information will begin to be returned to a subset of patient-participants later in 2018 and the COMPASS™ PG× report will be deployed at that time. For both new reports, we plan to capture access and navigation data to discern how the patients and providers use the report. In addition, we plan to contact patients and providers to obtain more detailed information about usability, accessibility, satisfaction and communication associated with the reports.

## Limitations

This research is limited by the number of participants in certain portions of the study, by possible confusion with physician letters in the usual care arm verses the enhanced PGR, and by the demographic population that Geisinger serves. When assessing the effectiveness of the PGR, we had fewer parents analyzing the enhanced PGR because we had fewer diagnostic findings to report than initially anticipated. This limitation is addressed by the mixed-methods study design that captured rich interview data enabling us to interpret the survey and understand access and satisfaction results in the proper context [15]. Another limitation may be a ceiling effect regarding the satisfaction, understanding and confidence reported at baseline in the usual care arm of the return of results process. Parents felt very informed and confident in handling their child’s condition. The summary letter of the visit with the geneticist may have been confused with the enhanced PGR, resulting in an increase in parents reporting access to the enhanced PGR and limiting our ability to detect change using survey measures [15]. Lastly, there is an inherent limitation introduced by the predominantly Caucasian population that resides in our rural service area and receive their health care at Geisinger. This could limit the generalizability of our study to other populations including those of more diverse racial and ethnic backgrounds, and those residing in urban settings. A recent Community Health Needs Assessment [26] reported that approximately 86 percent of the population in the three main counties served by Geisinger in the Northeast are White. The percentage of the population in the Northeast with a Bachelor’s degree or higher is 23.6 percent versus 31 percent for the US population. While this may lead one to presume a lower health literacy in this region of the country, this may be mitigated by a strong desire for a diagnosis. Many of our study participants have been on a diagnostic odyssey searching for an explanation for their child’s symptoms for many years. This may lead to an increased health literacy within our cohort, as well as representing a cohort motivated to participate in research in an attempt to help their child [10].

## Major Themes

Our research focused on a patient-centered clinical decision support (PCCDS) initiative to enhance patient and provider understanding of genomic test results, facilitate appropriate follow-up care, improve communication and increase overall satisfaction with use of genomic information. The formative work that preceded development of the COMPASS™ genomic report identified the need for supporting information about genomic results and proposed key elements to include in a results report. Direct patient involvement in the design and development of the PCCDS tool improved the ability to present comprehensive information about genomic sequencing results relevant to rare and often newly described diagnoses in the case of the PCORI study. Patients also reported that the CDS-derived genomic result report contributed to enhanced shared decision making, increased patient empowerment, and instilled greater confidence with their child’s rare condition. The patient perspectives gathered to inform the report content and deployment were essential to creation of the CDS system and led to the inclusion of important information that was deemed ‘too complex and detailed’ by researchers and providers but was highly valued by the patients.

While CDS tools have been studied and used for years, there is more work to be done to understand their full potential, in particular the role of patient facing CDS which has lagged behind systems directed at providers. Future research is needed to substantiate and further develop the relationship between patient-facing CDS and patient reported outcomes of importance. We demonstrated that substantial patient involvement at the initiation of this research contributes to improved design and facilitates earlier recognition of the outcomes that matter most to patients.

There is the opportunity to continue to adapt the COMPASS™ genomics report to meet additional clinical needs in situations that require transmission of complex data to patients in a supported, functional environment. PG× results can inform risk for adverse events, drug choice and dosing, but are generally only relevant when a patient is prescribed a medication. This can occur at any time after the testing occurs, or may never happen if the patient doesn’t require the medication. We found the PGR was well-suited to convey pharmacogenomic results and other genomic sequencing results. There is potential for use in the case of reporting somatic genetic testing results from tumor testing in Oncology. The important element in any future adaptation will involve talking with patients most likely to be impacted by the results to be reported in order to draft the optimal content, format and delivery of the information.

The COMPASS™ tool uses a standard application interface that can be associated with different EHR environments and therefore could be supported at institutions other than Geisinger. As more sophisticated standard interfaces are developed, (such as Fast Healthcare Interoperable Resources or FHIR), the COMPASS™ tool can be adapted to lower barriers to implementation in standards-compliant EHR systems.

## Conclusion

Patients contributed important insights into content, presentation, functionality and acceptability in the development of a patient-centered CDS-tool, the COMPASS™ genomic results report. Patients and our patient investigator made significant improvements in the tool as initially conceived, but also facilitated trouble-shooting as the tool was implemented. Their active investment in this project makes this CDS both patient-developed and patient-centered. Research and development of patient-centered CDS tools designed to support improved patient outcomes, are enhanced by early and substantial engagement of patients in contributing to all phases of tool design and development.

## Human Studies and Informed Consent

All procedures followed were in accordance with the ethical standards of the responsible committee on human experimentation (institutional and national) and with the Helsinki Declaration of 1975, as revised in 2000. This research was reviewed and approved by the Geisinger Institutional Review Board (IRB#: 2013-0594) as a study within a study with completion of the baseline survey as acceptable consent to participate.
